# Supplementary material for: The Intake of Ultra-Processed Foods and Prevalence of Chronic Kidney Disease: The Health Examinees Study
Source: Nutrients. 2022 Aug 28;14(17):3548. doi: 10.3390/nu14173548 (PMC9460585; doi:10.3390/nu14173548)
Supplement: Supplementary file 1 [file nutrients-14-03548-s001.zip › nutrients-1848476-supplementary.pdf]

Supplementary Table S1. Classification of FFQ food items according to the NOVA classification

NOVA group	HEXA Food group on the FFQ	Examples of food items/recipe items
1) Unprocessed or minimally processed foods	White rice ; multi-grain rice; mixed-grain rice; bean rice; mixed rice (half bean half rice); rice cake	White rice, buckwheat, soybean, black bean, black pollack, seoritae, Gijang, fermented soybean, sorghum, tea water, Yulmu rice, glutinous rice, glutinous rice cake , tteok, rice balls
	**Dumpling**	Tofu*, flour*, pork*, tenderloin*, boiled bean sprouts*, raw eggs*
	**Black Bean Noodles/Spicy Seafood Noodles**	Raw onions*, pork*, raw carrots*, beans*, raw mushroom*, raw garlic*, raw squid*, mussels*
	**Peanuts/almonds/Pine nuts**	Dried peanut*, roasted peanuts*, roasted pine nuts*
	Beans	Soybean, black bean, black pollack; dried yellow bean, soy sauce
	**Fermented soybean paste**	Fermented soybean*
	eggs/quail eggs	
	**Tofu**; "muk"	Tofu; acorn jelly, Mung-bean cake,buckwheat cake
	Potatoes and sweet potatoes	Raw potatoes, raw sweet potatoes
	Radish; cabbage/cabbage soup; spinach; lettuce; perilla leaves; vegetable wraps/salads; other green vegetables; bellflower/duck; bean sprouts/host sprouts; bracken/sweet potato stems; oyster mushrooms; other mushrooms; red pepper leaves/chive/minari; green onion/pumpkin juice; pumpkin	
	**Carrot/carrot juice**	Raw carrot*
	Grilled pork belly; Grilled pork/Stir-fried/broiled; Steamed pork; By-products (beef entrails, blood pudding, blood sausage); steak/roasted beef (ribs, sirloin, tenderloin, bulgogi; dog meat; fried chicken/ chicken soup/ "samgyetang"/ chicken soup; soups (beef soup, "yukgaejang", etc.)	
	Raw fish	Flounder, Tuna, sea bass
	Raw mackerel / saury; hairtail; eel; corvina / sea bream/flounder	
	Pollack, dried; squid/octopus, dried; anchovies, stir-fried	Raw Octopus, raw flounder, raw pollack, frozen pollack, raw octopus, raw squid, raw anchovies
	clam/snail (including soup, stew, grilled, chopped noodles, seasoned, etc.)	Raw crabmeat, ramie clam, raw lily, raw clam, raw blood clam, raw mussels
	Oyster; Crab; Shrimp	Oyster, crab, sesame , shrimp(boiled, raw, fried)
	Seaweed	laver, raw seaweed, raw kelp

	**Milk**	Plain milk
	Coffee; green tea	Coffee powder, instant; green tea
	strawberry; melon/melon; watermelon; peach/plum; banana; persimmon/dried; tangerine; pear; apple; orange/orange juice; grape/wine juice; **tomato/tomato ketchup**	Strawberry; melon musk; melon; watermelon, raw; plum; peach, raw; banana, raw; persimmon, tangerine, raw; kumquat, raw; stone pear; pear, raw; apple, raw, apple juice, grape juice; tomato juice
2) Processed culinary ingredients	**Jam/Honey/Butter/Margarine (if eaten on bread)**	Honey*
	**Black Bean Noodles/Spicy Seafood Noodles**	Black soybean sauce*, soybean oil*, green pepper powder*
	Grain powder	Deodeok root powder*, lactic acid bacteria*
	Table sugar; coffee cream	Table sugar, coffee cream[a]
	**Dumplings**	soybean oil*
3) Processed foods	**peanuts/almonds/Pine nuts**	Seasoned almonds*
	Korean spaghetti	Boiled noodles, boiled Udon
	**Black Bean Noodles/Spicy Seafood noodles**	Boiled chinese noodles*, potato powder*
	Cold noodles	Raw buckwheat noodles
	Glass noodles/potato noodles	
	Fermented soy-bean paste	Mixed paste ("ssamjang")*
	**Tofu**	Soft tofu*
	Pickled vegetables	Pickled red pepper, Pickled radish, Pickled garlic
	Grain powder/pre-meal (ready to eat porridges)[a]	barley, powder; sesame, black sesame, roasted; kelp, dried; carrot, raw; minari; "Myeongil" leaf; radish; Seaweed, powder; white rice; sorghum; mugwort, raw; lotus root; mushroom, raw; burdock, raw; yulmu rice; kale; shiitake mushroom, oak, dried; dried pumpkin
	corvina / sea bream / crabmeat; Pollack; dried squid / octopus; anchovies / stir-fried anchovies ; tuna-can; salted fish; fishcake / crabmeat; seaweed / kelp	Tuna-can; anchovy, salted; pollack, salted; yellowtail, salted; sea urchin, salted; squid; salted; shellfish; shrimp, salted, fried fish cake; dried seaweed

	Cheese	Processed cheese; mozallera cheese[a]
	**Other drinks**	Citron tea*, Ginseng tea*, "Ssanghwa" tea*
	carrot/carrot juice; peach/plum; apple; orange/orange juice; grape/wine juice; **tomato/tomato juice/tomato ketchup**	carrot can juice; peach, canned; orange, canned juice; tomato, canned tomato; tomato, tomato paste, canned tomato; tomato puree
	Fermented vegetables ("Kimchi")	Cabbage kimchi, radish kimchi, "Nabak kimchi/Dongchimi" and other kimchi
4) Ultra-processed foods	Instant noodles ("ramen")	Instant noodles
	cornflakes	cereal, rice crisps; cereal cornflakes; cereal, brown rice flakes
	Loaf bread; bread with red bean; other bread	Bread, red bean bread, buns, red bean filling, gombo bread; doughnut; cream bread; castella
	**Jam/honey/butter/margarine (when eaten on bread)**	strawberry jam*, apple jam*, apricot jam*, grape jam*, butter*, margarine*
	Cake/chocopie; cookie/cracker/snack; candy/chocolate	Choco Pie, Roll Cake, Whipped Cream, Blueberry Cake, Sponge Cake, Pound Cake, Hot Cake/Pancake, Montshell /Fresh cream; Dry Bread, Biscuit, Soft, Sen Bay, Rice Cake, Potato chips/crisps, Shrimp Snack, Corn snacks, Crackers; Drops, Candy
	Pizza/hamburger	Pizza, hamburger
	Processed meat (ham, sausage)	Raw bacon, raw Sausage, dry Sausage, Frankfurt Sausage, Hot Dog, Ham, Slice, Luncheon Meat
	Fish cake/Crab meat	Imitation crab*, crab sausage*
	**milk**	Coffee milk*, chocolate milk*, fruit flavored milk*
	yogurt/Yoplait	Liquid yoghurt, strawberry-flavored yoghurt
	Ice-cream	Ice cream, 12% fat; ice cream, 8% fat; ice cream, strawberry-flavored; ice cream, vanilla flavored; ice cream, chocolate-flavored; sherbet; selection; world corn; Joanna
	Soymilk drink	Soymilk drink (vegemil)
	Soft drinks (cola/cider) and fruit sodas	fruit soda; lemon soda; orange soda; cream soda; grape soda; cider; coke
	**Other drinks**	Sweet rice punch ("Sikhye")*
	**tomato/tomato juice/tomato ketchup**	Tomato ketchup*

Food groups in bold contain food items with different degrees of processing. These food groups appear in more than one NOVA class.

*Calculated by applying weights (% contribution of the food item/recipe item to the overall food group or mixed dish).

[a] Classified as UPF in sensitivity analysis

Supplementary Table S2. Sensitivity analysis for the association of UPF intake and prevalence of chronic kidney disease

		Quartiles of UPF intake, % food weight			
		Q1	Q2	Q3	Q4
No diabetes, hypertension or CVD	Cases	1,109	868	847	831
	PR (95% CI)[a]	1.00	0.95 (0.83-1.09)	1.16 (1.02-1.33)	1.19 (1.05-1.36)
Nutrient adjusted model	Cases	1,566	1,272	1,341	1,359
	PR (95% CI)[b]	1.00	0.99 (0.92-1.07)	1.12 (1.04-1.21)	1.11 (1.03-1.21)
Complete case analysis	Cases	1,270	1,067	1,106	1,123
	PR (95% CI)[c]	1.00	0.99 (0.92-1.08)	1.10 (1.01-1.2)	1.12 (1.03-1.22)
Including Cheese, coffee cream and pre-cooked porridges in UPF calculation					
	Cases	1,566	1,272	1,341	1,359
	PR (95% CI)[c]	1.00	1.02 (0.95-1.1)	1.15 (1.06-1.23)	1.17 (1.08-1.26)

[a] Adjusted for age, sex and total energy intake, educational level, income level, employment, smoking, drinking, physical exercise, and BMI

[b] Further adjusted for total fat intake, dietary phosphorus and dietary cholesterol intake

[c] Adjusted for age, sex and total energy intake, educational level, income level, employment, smoking, drinking, physical exercise, and BMI , high blood pressure, high blood sugar and CVD.
